# Supplementary material for: Urinary Metals Concentrations and Biomarkers of Autoimmunity among Navajo and Nicaraguan Men
Source: Int J Environ Res Public Health. 2020 Jul 22;17(15):5263. doi: 10.3390/ijerph17155263 (PMC7432079; doi:10.3390/ijerph17155263)
Supplement: Supplementary file 1 [file ijerph-17-05263-s001.pdf]

Limits of Detection (LODs)		Navajo & Nicaraguan*	NHANES 11-12+
UAS3	Arsenous (III) Acid	0.12	0.48
UAS5	Arsenic (V) Acid	0.79	0.87
UASB	Arsenobetaine	1.16	1.19
UASC	Arsenocholine	0.11	0.28
UDMA	Dimethylarsinic Acid	1.91	1.8
UMMA	Monomethyl arsonic Acid	0.2	0.89
UTMO	Trimethylarsine oxide	0.17	0.25
UUR	Uranium	0.002	0.003
UTU	Tungsten	0.018	0.026
UTL	Thallium	0.018	0.02
USR	Storntium	2.34	2.5
USN	Tin	0.09	0.22
USB	Antimony	0.022	0.41
UPB	Lead	0.03	0.08
UMO	Molybdenum	0.8	0.99
UMN	Manganese	0.13	0.08
UCS	Cesium	0.086	0.12
UCO	Cobalt	0.023	0.48
UCD	Cadmium	0.036	0.056
UBA	Barium	0.06	0.1
*Sent with laboratory results by CDC<br>+Published by CDC [59].			

Supplementary Table 1. Limits of Detection
